# Comparison of ZrO_2_ Particles and Polyaniline as Additives in Polystyrene-Based Sorbents for the Micro-Solid Phase Extraction of Psychoactive Drugs from Biofluids

**DOI:** 10.3390/molecules29040761

**Published:** 2024-02-07

**Authors:** Paweł Stelmaszczyk, Mateusz Iwan, Dominika Pawcenis, Renata Wietecha-Posłuszny

**Affiliations:** 1 Laboratory for Forensic Chemistry, Department of Analytical Chemistry, Faculty of Chemistry, Jagiellonian University in Kraków, Gronostajowa St. 2, 30-387 Kraków, Poland; pawel.stelmaszczyk@doctoral.uj.edu.pl (P.S.); mateusz.iwan@alumni.uj.edu.pl (M.I.); 2 Doctoral School of Exact and Natural Sciences, Faculty of Chemistry, Jagiellonian University in Kraków, Gronostajowa St. 2, 30-387 Kraków, Poland; 3 Faculty of Chemistry, Jagiellonian University in Kraków, Gronostajowa St. 2, 30-387 Kraków, Poland; pawcenis@chemia.uj.edu.pl

**Keywords:** electrospun fibers, solid phase microextraction, sorbents, polystyrene, psychoactive substances

## Abstract

The intensive development of extraction methods based on μ-SPE extraction contributes to the increased interest in the synthesis of new sorption materials. This work presents the characterization of polystyrene fibers and polystyrene fibers blended with ZrO_2_ particles or polyaniline obtained by electrospinning and their use in the extraction of selected psychoactive drugs from biological samples. The characteristic of produced fibers is made by performing SEM images, measuring average fiber diameter, and examining their sorption abilities. Among the fibers based on pure polystyrene, tested in the first stage, the best sorption properties are demonstrated for the fibers obtained from a polystyrene solution in DMF with a concentration of 17.5 wt%. In the next stage, this material was modified with synthesized ZrO_2_ particles and polyaniline. Among the tested materials, the sorbent based on polystyrene with polyaniline shows the best sorption properties of the tested substances. The use of this material in the μ-SPE in a needle enables the extraction of selected compounds from aqueous and biological samples such as urine and human plasma.

## 1. Introduction

The most intensive development in the field of micro-solid phase extraction (μ-SPE) is currently carried out in the field of synthesis and production of new sorbent materials that can be used in the extraction process of a wide range of chemical compounds. Finding a material with the desired parameters and properties allows for increased selectivity and sensitivity of the analytical procedure. These improvements are achieved by raising the efficiency of the extraction process and better isolation of the analytes from a complex matrix. Useful materials in this area are micro- and nanomaterials, which, due to a larger specific surface area, often have better mechanical, thermal, and chemical stability, and an increased affinity ratio of analytes to the extraction phase, improving the efficiency of the extraction process [1,2]. Currently, many materials are used in solid-phase extraction techniques: carbon nanomaterials, nanostructured polymers, silica-based nanomaterials, and metal nanoparticles [1,3].

The polymer-based fibers are increasingly used in extraction techniques such as solid phase extraction (SPE) or solid phase microextraction (SPME). Although there are many known methods of producing fibers on the micro- and nanoscale, including template synthesis, melt blowing, and molecular self-assembly, electrospinning is the most commonly used technique [2,4]. In this process, a high voltage is applied to the polymer solution and the obtained fibers are collected on the collector [4]. The morphology and quality of the formed fibers are influenced by process parameters such as applied voltage, flow rate, the distance between the needle with solution and collector, and the parameters of solutions and environment. The optimization of these parameters leads to obtaining fibers with the desired morphology and diameter of even a few nanometers [5]. Depending on the properties of the tested analytes and the type of sample, it is possible to produce fibers based on a wide range of polymers with different properties. Polystyrene (PS), polyacrylonitrile (PAN), and polyamide 6 (PA 6) are frequently used polymers to produce the extraction phase by electrospinning [2]. Moreover, the electrospinning process enables the production of modified fibers, for example, by producing a material consisting of several polymers (blended polymers), material containing nanoparticles, or metal-organic frameworks (MOFs) [4].

Currently, micro- and nanofibers based on many different compositions have been used in different areas of analysis: food analysis [6,7], environmental purposes [8,9], and bioanalysis [10,11,12]. An example of the use of nanofibers in the sample preparation of biological samples was presented in the publication by An et al. [13] This study presented the application of SPE extraction with the use of a sorbent in the form of polystyrene nanofibers to determine eight sedative drugs (including zolpidem, diazepam, and lorazepam) in human urine. The detection limits of this method were in the range of 0.2–0.4 μg/mL in urine, while the intra-day precision of the determinations of tested drugs was in the range of 1.3–15.2%. Kandeh et al. [14] used nanofibers based on poly(vinyl alcohol)—PVA and poly(acrylic acid)—PAA blended with carbon nanotubes (CNT) and cellulose nanocrystals (CNC) (PVA-PAA/CNT-CNC) for the determination of seven opioids (including morphine, codeine, and tramadol) in human plasma. The obtained detection limits of the developed procedure were in the range of 0.15–50 ng/mL for all analytes. The intra-day and inter-day precision of determinations were 5% and 6%, respectively.

Several materials containing polyaniline (PAni) and zirconium(IV) oxide were characterized and used in different types of SPE configurations [15,16,17,18,19,20,21,22]. The PAni-based fibers were used for the isolation of selected triazines from aquatic samples using flow injection μ-SPE. The recovery of the proposed extraction was in the range of 83–95% with intraday RSD in the range of 8.9–9.5% [15]. The use of blended sorbent based on PAni and PAN was reported in the case of environmental waters and food sample preparation for the determination of hydrophilic non-steroidal anti-inflammatory drugs [20] or fluoroquinolones [19]. Moreover, PAni-based fibers blended with graphene oxide were used in the extraction of metal ions Pb^2+^ from beverage samples with high repeatability and reproducibility [17]. Conducting polymers with particular emphasis on polyaniline are a promising class of substrates for sorbent materials. Due to PAni protonation reversibility, Pani-based sorbents can selectively bind to or release a molecule of interest just by selecting the proper pH of a solution/elution buffer. To date, most applications of PANI in sorbent materials cover the removal of heavy metal or organic dyes from water [23], whereas the extraction of drugs from biological samples seems still moderately explored.

The modern development of µ-SPE extraction focuses on miniaturized and automated processes that reduce the consumption of organic solvents. The variety of available sorption materials makes it possible to develop a material that is selective for specific analytes. Polystyrene shows great potential for use in the isolation process of many biologically active substances from polar samples, e.g., water or biological samples. The materials with a high specific surface area are most desirable. The fibers doped with, e.g., metal oxides, lead to an increase in the specific surface of the material, which may contribute to increasing the effectiveness of the isolation of psychoactive substances from the investigated samples. The presented study was aimed at examining the possibility of using a sorbent material based on polystyrene doped with zirconium(IV) oxide particles or polyaniline to increase sorption properties by increasing the specific surface of bare polystyrene fibers. For cost-saving, we propose synthesis protocols for both the components used. The production of fiber components makes it possible to reduce the costs of obtaining them in relation to the price of the manufactured reagent purchased from the manufacturer. The material with the best sorption properties was used to extract selected psychotropic drugs from biological samples of human urine and plasma.

## 2. Results and Discussion

### 2.1. Characterization of ZrO_2_ Particles

The X-ray diffraction pattern of synthesized ZrO_2_ particles (*λ* = 1.5405 Å, Bragg-Brentano geometry, 2*θ* range: 5–90°) and scanning electron microscopy (SEM) image are included in Supplementary Materials in Figure S1A,B.

The obtained crystalline diffraction peaks at 2*θ* values of 28.2°, 30.3°, 31.5°, 35.3°, 38.5°, 41.5°, 50.3°, and 60.1° match the monoclinic form of ZrO_2_ [24,25].

The average crystalline diameter was calculated with the use of the Scherrer equation (Equation (1)):

(1) $$D_{hkl}=\frac{K\cdot \lambda }{B\left(2\theta \right)\cdot \cos(\theta )}$$

The following notations in Equation (1) stand for: $D_{hkl}$ is the average diameter [nm], *K* is a shape factor (*K* = 0.9), *λ* is the wavelength [nm], *θ* is a diffraction angle [°], and $B\left(2\theta \right)$ is the full width at half maxima of a diffraction peak [rad]. The values of the diameter obtained for the most intense peaks are summarized in the Supplementary Materials in Table S1.

The average crystallite diameter of ZrO_2_ particles was 15.25 nm, proving the formation of ZrO_2_ nanoparticles. From the SEM images (Figure S1B), it can be seen that ZrO_2_ grains tend to form bigger lumps; however, this could be ascribed to the quality of sample grinding in the mortar.

**Figure 1:**
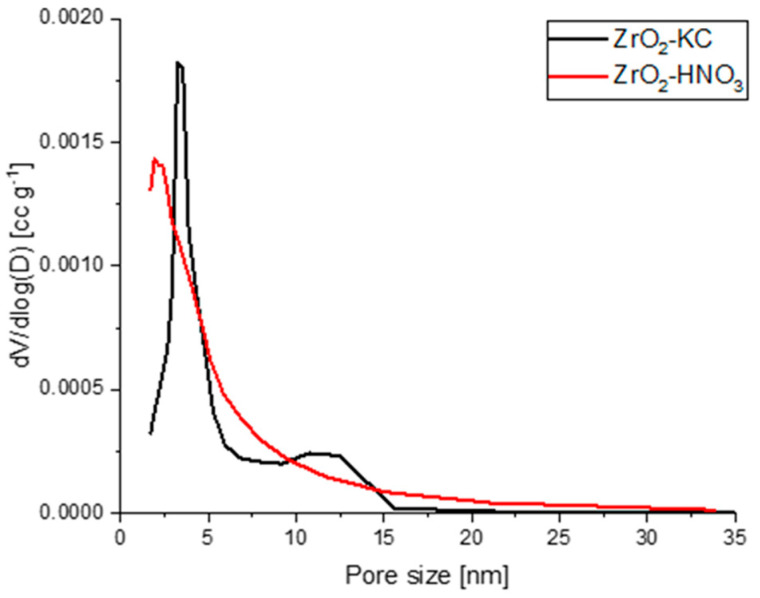
Pore size distribution of calcined ZrO_2_ (ZrO_2_-KC) and ZrO_2_ washed with nitric acid (ZrO_2_-HNO_3_).

Before preparing solutions for electrospinning, ZrO_2_ particles were purified from citric acid residues by calcination (ZrO_2_-KC) or by washing with HNO_3_ (ZrO_2_-HNO_3_). The specific surface area for ZrO_2_-KC was equal to 39.85 m^2^/g, whereas for ZrO_2_-HNO_3_ it was 76.30 m^2^/g. The detailed values for SSA, pore volumes, and sizes are given in Table 1, whereas pore size distributions are presented in Figure 1.

It can be seen that in both samples, mesopores prevail, as the determined values are within the range of 2–50 nm according to the IUPAC classification [26]. In the case of sample ZrO_2_-KC, most of the pores are characterized with a diameter of 3.32 nm, whereas a less abundant fraction of pores is also found between 10 and 15 nm. Sample ZrO_2_-HNO_3_ is characterized by lower average pore diameter, higher average pore volume, and higher specific surface area. The main fraction in the pore size distribution curve is ascribed to the pores with a volume of 2 nm.

### 2.2. Characterization of PAni

**Figure 2:**
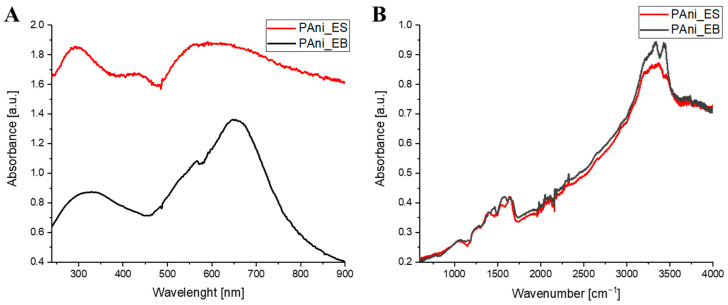
(**A**) UV-Vis spectra of both forms of PAni: emeraldine salt (PAni_ES) and emeraldine base (PAni_EB); (**B**) ATR-FTIR spectra of both forms of PAni.

The synthesis of polyaniline emeraldine salt (PAni_ES) and base (PAni_EB) was confirmed by collecting UV-Vis (Figure 2A) and ATR-FTIR (Figure 2B) spectra. The bands visible in the UV-Vis spectrum for the polyaniline emeraldine salt (at approximately 330 and 660 nm) and the polyaniline emeraldine base (320 and 620 nm) coincided with the data available in other publications [27,28]. The ATR-FTIR spectra show bands resulting from the N-H stretching vibrations at around 3445 cm^−1^, the C=N stretching vibration at around 2325 cm^−1^, the C=C stretching vibration of the benzenoid ring (at around 1560 cm^−1^), the quinine ring (at around 1470 cm^−1^), and the C-N stretching vibration at around 1278 cm^−1^.

### 2.3. Characterization of PS-Based Fibers

**Figure 3:**
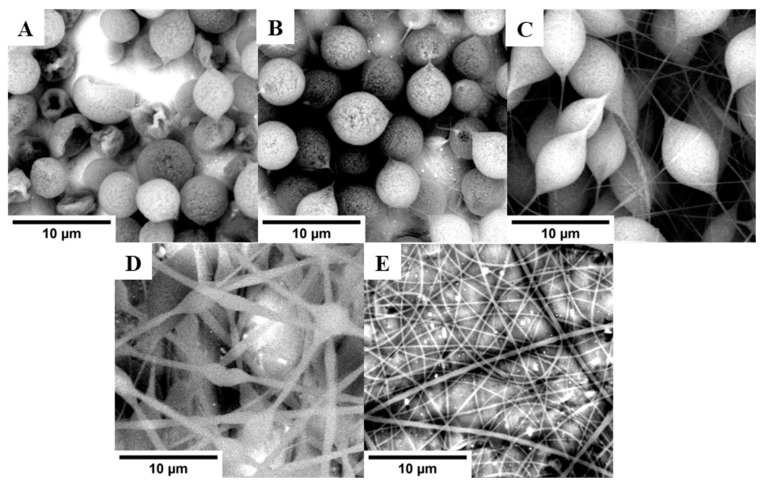
SEM images of the PS-based fibers obtained by electrospinning solutions at different concentration levels of PS: (**A**)—10.0 wt%; (**B**)—12.5 wt%; (**C**)—15.0 wt%; (**D**)—17.5 wt%; (**E**)—20.0 wt%.

The pure PS-based materials prepared in the electrospinning process were characterized by SEM images and analyzing the diameter distribution of the obtained fibers. During the presented research, the focus was placed on determining parameters directly influencing the sorption properties of the materials (such as the pore size of ZrO_2_ described in Section 2.1, average fiber diameter). To determine their potential applications in other fields, an extended mechanical characterization of the materials would be necessary, including, e.g., tensile strength, elasticity, and Young’s modulus. Figure 3 presents SEM images for materials obtained from PS solutions in DMF at a concentration in the range of 10–20 wt%. The average diameters of PS fibers are summarized in Table 2. The increase in PS concentration in the solution caused a decrease in the number of beads in the structure and the average diameter of fibers. For a DMF solution of PS at the concentration of 10.0 and 12.5 wt%, the structure of the obtained material consists mostly of beads. The formed fibers were more visible at a higher concentration of PS. The best structure of the pure PS-fibers was obtained for the PS at a concentration of 20.0 wt%. This material was also characterized by the lowest fiber diameter.

The µ-SPE procedure was performed to characterize the sorption properties of PS-based fibers. The standard solution of tested substances (ketamine, flunitrazepam, diazepam, nitrazepam, temazepam, lorazepam, desipramine, amitriptyline, imipramine, paroxetine, citalopram, cocaine, norcocaine, and cocaethylene) was analyzed by using all fibers as the sorption phase in the µ-SPE process. Sorption properties were assessed based on the value of the F function. Equation (2) presents the formula of the evaluation function.

(2) $$F=2.5\cdot \bar{\alpha }\;\cdot \sum_{i=1}^{n}1.05^{\left(\frac{\alpha_{i}}{100}\right)}\cdot \prod_{i=1}^{n}1.2^{-(\frac{\beta_{i}}{10})}$$

The evaluation function takes into account the following parameters: $\alpha_{i}$—signal ratio for the analyte in the sample after the µ-SPE process, and the sample not subjected to the process; $\bar{\alpha }$—the average value of the *α* parameters for the tested material; $\beta_{i}$—the quotient of the standard deviation and the mean value of the *α* parameter for each analyte. The function and its parameters have been fine-tuned to promote materials capable of detecting as many test drugs as possible while also maintaining good precision while strongly reducing the function value in the event of not detecting one of the compounds and a large scatter of results.

Comparing the diameter of the PS-based fibers with the determined values of the F function, it seemed that materials with a lower diameter of the fibers had better sorption properties than the tested drugs. However, the highest value of the F function was determined for fibers obtained from PS solution at a concentration of 17.5 wt%. Better results for PS 17.5 wt% (with a wider diameter of fiber than for PS 20.0 wt%) may be caused by failure to adapt the parameters of µ-SPE extraction procedure for efficient condition and desorption for the PS 20.0 wt% material. Another explanation for these results may be the difference in the pore diameters of both tested materials. This issue may be explored in further research by changing the extraction procedure. Despite this, the result obtained for PS 17.5 wt% was found to be satisfactory, and it was decided that this material would undergo the next stage of the presented research.

**Figure 4:**
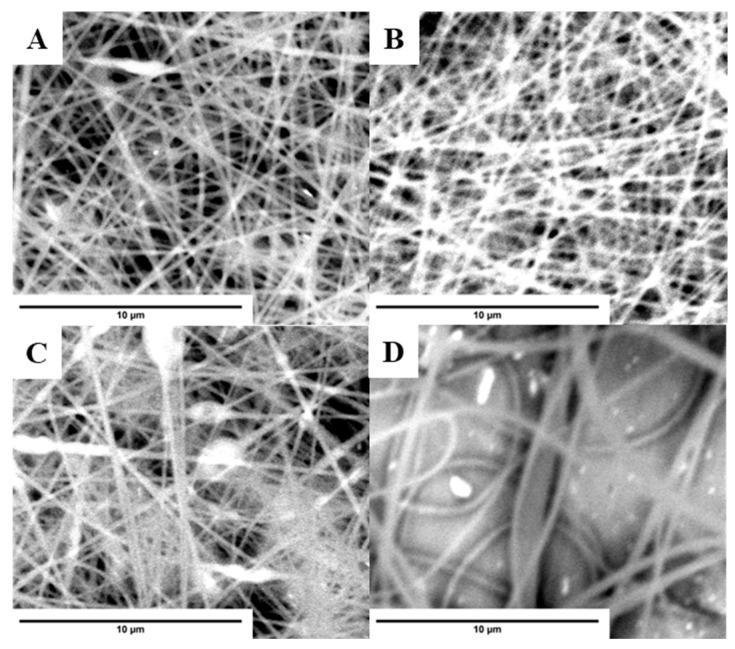
SEM images of the modified PS 17.5 wt% fibers obtained by electrospinning: (**A**)—PS 17.5% + PAni_ES; (**B**)—PS 17.5% + PAni_EB; (**C**)—PS 17.5% + ZrO_2_-KC; (**D**)—PS 17.5% + ZrO_2_-HNO_3_.

**Figure 5:**
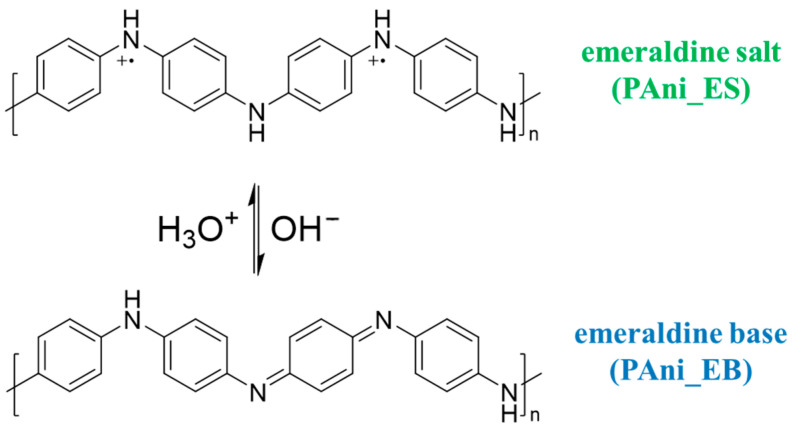
Structures of two forms of PAni: PAni_ES and PAni_EB.

In the next step, the following blended materials based on PS 17.5 wt% were produced and characterized with polyaniline emeraldine salt (PS 17.5% + PAni_ES), polyaniline emeraldine base (PS 17.5% + PAni_EB), calcined ZrO_2_ particles (PS 17.5% + ZrO_2_-KC), and ZrO_2_ particles purged with nitric acid (PS 17.5% + ZrO_2_-HNO_3_). These materials were characterized by SEM images and by analyzing the diameter distribution of the obtained fibers. The sorption properties of these materials were also assessed based on Equation (2). The summarized characterization of the obtained fibers is presented in Table 2 and Figure 4. The thinnest fibers (0.2 µm) were obtained for the PS 17.5% + PAni_ES material. This material had the best sorption properties of the tested drugs from water samples. This result can be explained by the possibility of various types of analyte–polyaniline interactions resulting from the PAni structure. Figure 5 shows the structures of two forms of PAni: PAni_ES and PAni_EB. Aromatic rings enable π-π interactions with molecules that have an aromatic system, which contains many organic molecules that are psychoactive substances. Moreover, the presence of hydrogen atoms in the -NH- group makes it possible to form hydrogen bonds between the material and the analyte. Additionally, charged ionic groups with a positive charge may appear in the structure of polyaniline, enabling the formation of electrostatic bonds with anions. Therefore, the structure of both PAni_ES and Pani_EB forms enables the formation of hydrogen interactions and π-π interactions with a wide range of various psychoactive substances. Moreover, PAni_ES enables the occurrence of additional electrostatic interactions, which can explain the better sorption capacity of the material produced on its basis. Based on the results, it can be seen that the addition of PAni_EB and ZrO_2_-KC significantly decreased the sorption properties of pure PS 17.5 wt% material. However, the addition of ZrO_2_-HNO_3_ particles increased the extraction potential of the tested analytes from water samples. It can be assumed that the use of nitric acid to purify the synthesis product was more effective than the calcination of ZrO_2_. However, these differences may also result from the different specific surface area and pore diameter of the oxide, which was purified in two different processes before being blended with polystyrene. ZrO_2_ washed with nitric acid is characterized with SSA twice the surface area of sample ZrO_2_ calcinated. The resulting pore volumes for ZrO_2_-HNO_3_ are somewhat bigger; thus, the active surface for analytes adsorption is also higher.

### 2.4. Biological Samples Analysis

The PS 17.5 wt% + PAni_ES material was tested in the case of extraction of fourteen substances (including ketamine, benzodiazepines, antidepressants, and cocaine) from urine and plasma samples. This material was selected based on the characterization presented in the previous paragraph: the smallest diameter of fibers and the best sorption properties. The possibility of its use in µ-SPE extraction to isolate analytes from biological samples (urine and plasma) was tested. The precision of determinations was determined based on the analysis of three independent samples at the concertation of 100 ng/mL of each analyte.

Table 3 presents the calculated precision of determinations. The precision for urine samples has not exceeded 10% for most tested drugs. The worst precision was obtained for imipramine (CV% = 12.6%). However, the precision for all analytes has not exceeded the value of 15%, which is a satisfactory precision in toxicological analyses. In general, the values of precision for plasma samples were higher for all analytes. It should be emphasized that this may be influenced by a more complicated composition of the biological sample matrix. The deproteinization stage used in the sample preparation for the extraction process could also have an impact on the obtained results. Additionally, the samples were not degreased, which might have affected the results. Moreover, three analytes (amitriptyline, paroxetine, and, citalopram) could not be detected in the extracts. It could be caused by the presence of a matrix effect or the failure to isolate them from the matrix at the sample deproteinization stage. Moreover, the high values of CV% for ketamine in plasma or imipramine in urine could be reduced via the modification of the extraction procedure involving the washing buffer solutions for conditioning the PAni bed before the extraction. The pH of the conditioning solution may influence the structure of PAni, leading to variations in the absorption of amine compounds such as ketamine and imipramine. This issue should be taken into account for the future optimization of extraction procedures using this material.

The preliminary experiment of biological sample analysis indicates the successful use of the selected PS 17.5% + PAni_ES material in the analysis of biological samples. It should be emphasized that to use this material in routine µ-SPE extraction, it is necessary to optimize the entire sample preparation process, including testing and measuring the absorption and desorption curves of the tested analytes, which may additionally improve the validation parameters of the analytical method using µ-SPE extraction with characterized material.

## 3. Materials and Methods

### 3.1. Chemicals and Materials

The following chemicals and materials were used during the experiments. Ultra-pure water was obtained via purification by the Milli-Q Plus system (Merck-Millipore, Darmstadt, Germany). Ammonium hydroxide (28–30%), absolute ethanol (>99.9%), zirconyl chloride (>98%), acetic acid (>99.9%), synthesis-grade aniline, ammonium persulfate (>98%), acetone (>99.5%), polystyrene (average molecular weight, Mw~192,000), dimethylformamide (>99.8%), N-methyl-2-pyrrolidone (>99%), formic acid (98–100%), perchloric acid (70%), methanol (>99.9%), and acetonitrile (>99.9%) were purchased from Sigma-Aldrich (St. Louis, MO, USA). Citric acid monohydrate, analysis-grade nitric acid, and analysis-grade hydrochloric acid were provided by POCH (POCH, Gliwice, Poland).

Standards of analytes in methanol: ketamine, flunitrazepam, diazepam, nitrazepam, temazepam, lorazepam, desipramine, amitriptyline, imipramine, paroxetine, citalopram, cocaine, norcocaine, and cocaethylene; and deuterated standards of chosen analytes: amitriptyline-D3, nitrazepam-D5, diazepam-D5, temazepam-D5, flunitrazepam-D3, and lorazepam-D4 were purchased from Lipomed AG (Arlesheim, Switzerland).

The drug-free biological samples were used during this study. The human plasma was purchased from a local blood bank (Cracow, Poland) and the human urine was obtained from a healthy volunteer who was not taking tested drugs. The biological samples were stored in a freezer at −20 °C.

### 3.2. Synthesis of ZrO_2_ Particles

ZrO_2_ particles were synthesized using a sol-gel method. First, 24 mL of 5 M ammonia solution and 8.4 g of citric acid monohydrate were mixed in a beaker. Then, 40 mL of 30% ethanol solution was added to the beaker. In another beaker, 16.5 g of zirconyl chloride octahydrate was weighed out and dissolved in 80 mL of 30% ethanol solution. The contents of both beakers were stirred for 30 min at a speed of 400 RPM. Afterward, the zirconyl chloride solution was added dropwise to the other solution. The combined solution was heated to about 70 °C for an hour and then transferred to a crystallizer and dried in a furnace at 50 °C. One part of the obtained product was then calcined at 400 °C for 6 h (ZrO_2_-KC). Another part of the dried ZrO_2_ was treated with concentrated nitric acid. When the reaction was finished, the content was transferred to a beaker filled with ethanol and then dried at 50 °C (ZrO_2_-HNO_3_). The last part was not treated in any way (ZrO_2_).

### 3.3. Synthesis of PAni

Polyaniline was synthesized using the following method. First, 18 mL of 2 M hydrochloric acid and 2 mL of aniline were placed in a beaker and stirred at a speed of 300 RPM. In another beaker, an aqueous solution of ammonium persulfate was prepared by the dissolution of 0.439 g of APS in 10 mL of ultra-pure water. Afterward, the APS solution was added dropwise to the aniline solution. The solution was then stirred for 30 min. Next, the solution was filtered through a glass sinter covered with filter paper, washed twice with 25 mL of a 0.2 M hydrochloric acid solution, and later with 20 mL of acetone. Then, the filter paper with a precipitate was put into a furnace and dried at 50 °C. This resulted in the creation of a dark green product—polyaniline emeraldine salt (PAni_ES). Part of the product was treated with diluted ammonia to obtain a dark blue product—polyaniline emeraldine base (PAni_EB).

### 3.4. Characterization of Synthesis Products

The synthesized ZrO_2_ particles were characterized by performing an XRD measurement using an X’Pert Pro MPD X-ray diffractometer (Phillips, Amsterdam, The Netherlands) with Johansson monochromator (line K_α1_ for Cu, λ = 1.5405 Å) and an X’Celerator silicon detector, and the SEM imaging was carried out using an SEM Phenom XL microscope (Thermo Fisher Scientific, Waltham, MS, USA). The polyaniline was characterized by the measurement of UV-Vis and ATR-FTIR spectra using an AvaSpec-ULS3648 spectrometer with a deuterium-halogen lamp (Avantes BV, Apeldoorn, The Netherlands) and FTIR Thermo Nicolet 8700 spectrophotometer with a DTGS detector (Thermo Scientific, Bremen, Germany) with GladiATR attachment (Pike Technologies, Cottonwood, WI, USA), respectively. 3Flex instrument (Micrometrics, Ottawa, ON, Canada) was used to record nitrogen isotherms at 77 K. Samples were degassed at 150 °C for 20 h before recording the isotherms. The specific surface area, pore size, and average pore volume of the samples were evaluated from nitrogen adsorption isotherms at 77 K, using the Brunauer–Emmett–Teller (BET) equation.

### 3.5. Preparation of Solutions for Electrospinning

The solutions for electrospinning were prepared by mixing adequate amounts of polystyrene with a solvent and, optionally, zirconia or polyaniline, and by stirring the solutions for at least 24 h at a speed of 400 RPM. The contents of the prepared solutions were as follows: 1–5 contained 10.0; 12.5; 15.0; 17.5; 20.0 wt% of PS in DMF; 6–17.5 wt% PS with ZrO_2_-KC in DMF; 7–17.5 wt% PS with ZrO_2_-HNO_3_ in DMF; 8–17.5 wt% PS with PAni_ES in DMF:NMP (1:1; *w*/*w*); 9–17.5 wt% PS with PAni_EB in DMF:NMP (1:1; *w*/*w*).

### 3.6. Electrospinning Process

The electrospinning process was carried out using the electrospinning equipment Fluidnatek LE-50 (Bioinica, Valencia, Spain) with the atmosphere control module. The process was performed under the same conditions for each prepared solution. The conditions were as follows: the voltage difference was 17 kV, and the distance between the needle and the collector was 18 cm. The solutions were pumped with a flow of 0.5 mL per hour. The temperature was kept at around 35 °C with relative humidity below 40%. The fibers were spun for 30 min each and collected on an aluminum foil.

### 3.7. Characterization of Fibers

The fibers were characterized by SEM imaging carried out with an SEM Phenom XL microscope (Thermo Fisher Scientific, Waltham, MA, USA). The diameter distribution of the obtained fibers was performed using ImageJ version 1.8.0_172 (LOCI, University of Wisconsin).

### 3.8. Preparation of Spiked Biological Samples

The biological matrices were spiked with tested substances according to the following procedure. First, the intermediate solution of each substance at the concentration of 10 µg/mL in methanol was prepared from the stock solutions. Next, the mixtures of all analytes and internal standards at a concentration of 500 ng/mL in methanol were prepared separately by diluting the intermediate solutions. To prepare spiked plasma and urine samples, the appropriate amount of mixtures of analytes and internal standards were dried at 45 °C in an Eppendorf vial, and next, the biological matrixes were added to the residue to obtain the final concentration of 100 ng/mL of each analyte and internal standard in the biological samples. The samples were prepared one day before the analysis and stored at −20 °C.

Before the analysis, plasma samples were deproteinized with 70% perchloric acid (200 µL of acid per 200 µL of plasma). The samples were mixed and centrifuged (5 min, 14,500 RPM). Next, the supernatant was analyzed. The urine samples were subjected to extraction without additional preparation.

### 3.9. Preparation of μ-SPE Needles

The sorbents (in the form of fibers) for performing the μ-SPE process were packed in injection needles (dia. 0.8 × 40 mm, 21G, KD-FINE, KDM, Czechowice-Dziedzice, Poland). The process of preparing needles was based on the review of the literature [29] and was as follows. The fibers were collected from the aluminum foil by using a spatula. From the collected fibers, (5.0 ± 0.1) mg of sorbent was weighed directly in the needle. Finally, the sorbent was tamped. The prepared needles were secured by parafilm to avoid any contamination till the extraction was performed.

### 3.10. μ-SPE Extraction

The extraction process was carried out using a syringe pump (Model 601553, KD Scientific, Holliston, MA, USA), plastic syringes with a volume of 2 mL, and μ-SPE in needles prepared according to the protocol described in the previous section. The first stage of the procedure was conditioning the sorbent with 50 µL of ethanol and 100 µL of ultrapure water. Next, the adsorption process was performed with the use of 100 µL of samples (standard solution in water or prepared biological samples). After adsorption, 50 µL of ultrapure water was pressed through the sorbent for purification. The desorption process was carried out using methanol. At this stage, the first drop of solvent that passed through the needle was thrown out and the next eight drops were collected in the insert located in the 1.5 mL HPLC vial. The flow rate of the solvent at each stage of the extraction procedure was 1 mL per hour. The collected part of methanol was evaporated at 45 °C. Next, 50 µL of 0.1% formic acid in ultrapure water was added to the residue in the insert and vortexed at the speed of 2500 RPM.

### 3.11. LC-MS Conditions

The chromatographic process was carried out using the UltiMate 3000 RS liquid chromatography system (UHPLC; Dionex, Sunnyvale, CA, USA) with Hypersil Gold Phenyl column (50 mm × 2.1 mm ID, particles 1.9 μm; Thermo Fisher Scientific, Bremen, Germany). The mass spectrometer MicroTOF-Q II from Bruker (Bremen, Germany) with an electrospray ionization source (ESI) was coupled with the chromatography system.

The chromatographic conditions and mass detector settings were chosen based on the previously developed method for psychoactive substances [30]. The mobile phase consisted of 0.1% formic acid in ultrapure water (eluent A) and acetonitrile (eluent B). The gradient program of the mobile phase during the single analysis was as follows: 0.0–4.0 min—eluent B increased from 15% to 40%; 4.0–7.0 min—eluent B remained constant at 40%; 7.0–10.0 min—eluent B increased to 70%; 10.0–12.5 min—eluent B decreased to 15%; 12.5–17.0 min—eluent B was held at 15% to stabilize the column prior the next injection. The flow rate of the mobile phase and the column temperature were set to 0.3 mL min^−1^ and 35 °C during the entire measurement, respectively. The volume of the injected sample was 5 µL.

The mass detector parameters were as follows. The capillary voltage and the nebulizer pressure were 4.5 kV and 2.5 bar, respectively. The dry gas flow and temperature were set to 5.5 L/min and 200 °C, respectively. The detector operated in the positive ionization mode in the scanning mode in the range of 50–800 *m*/*z*. The values of [M + H]^+^ ions of the tested substances and internal standards (IS) were extracted from the recorded chromatograms.

## 4. Conclusions

The electrospinning made it possible to easily obtain fibers with different morphology and fiber diameters. Depending on the composition of the electrospinning solution, it is possible to obtain fibers of different morphology. The results showed that the fibers obtained from the 17.5 wt% PS in DMF solution were characterized by the best sorption properties among the other tested PS-based materials. Additionally, procedures for the synthesis of ZrO_2_ particles and polyaniline were developed, and the obtained products were used to modify the polystyrene fibers. The materials with the best sorption properties were materials with the addition of polyaniline in the form of emeraldine salt (PAni_ES) and ZrO_2_ particles purified with nitric acid(V) (ZrO_2_-HNO_3_). Additional tests were carried out for the PS 17.5% + PAni_ES material to check the possibility of using it in the analysis of biological samples—urine and human plasma. The µ-SPE extraction with the use of this material enabled the extraction of chemical substances using different structures (including ketamine, benzodiazepine derivatives, antidepressants, and cocaine) from urine samples with satisfactory precision. Some substances were not detected in the extraction from human plasma samples, which may be related to the need to perform the deproteinization process before the extraction, which could influence the efficiency of the extraction process of these substances. However, the number of substances that could be isolated with satisfactory precision from both biological materials indicated a high potential for the use of the tested materials in toxicological analyses.

## Figures and Tables

**Table 1:** Specific surface area and pore size and volumes of ZrO_2_ samples.

Sample	SSA (m^2^/g)	Average Pore Size (nm)	Average Pore Volume (cm^3^/g)
ZrO_2_-KC	39.85	5.018	0.0547
ZrO_2_-HNO_3_	76.30	4.013	0.0765

**Table 2:** Parameters of the tested PS-based fibers.

Electrospinning Solution Composition	Average Fiber Diameter (µm)	F Value
PS 10.0%	4.38	12.6
PS 12.5%	3.86	14.9
PS 15.0%	2.39	30.6
PS 17.5%	1.01	40.3
PS 20.0%	0.26	34.4
PS 17.5% + PAni_ES	0.20	49.6
PS 17.5% + PAni_EB	0.25	25.4
PS 17.5% + ZrO_2_-KC	0.23	21.4
PS 17.5% + ZrO_2_-HNO_3_	0.44	48.4

**Table 3:** The precision of determination of selected substances at the concentration of 100 ng/mL in urine and plasma samples by µ-SPE extraction with PS 17.5% + PAni_ES fibers as a sorption phase.

Analyte	Precision—CV (%) (*n* = 3)	
	Urine	Plasma
Ketamine	3.7	26.0
Flunitrazepam	4.9	9.4
Diazepam	2.7	1.8
Temazepam	3.9	5.0
Nitrazepam	3.2	14.5
Lorazepam	2.6	11.6
Amitriptyline	6.5	not found ^1^
Imipramine	12.6	6.9
Desipramine	9.6	15.1
Paroxetine	9.3	not found ^1^
Citalopram	5.3	not found ^1^
Cocaine	2.7	6.0
Norcocaine	6.1	13.7
Cocaethylene	5.4	12.7

^1^ not found—analyte did not detect the tested concentration in a biological sample.

## Data Availability

Data are contained within the article and supplementary materials.

## Supplementary Materials

The following supporting information can be downloaded at: https://www.mdpi.com/article/10.3390/molecules29040761/s1, Figure S1: (A)—XRD pattern (*λ* = 1.5405 Å, Bragg-Brentano geometry, 2*θ* range: 5–90°) of synthesized ZrO_2_ particles; (B)—SEM image of obtained ZrO_2_ particles. Both XRD patterns and SEM images were obtained for ZrO_2_ particles before calcination or washing with HNO_3_; Table S1: The calculated diameter of synthesized ZrO_2_ particles based on the XRD pattern and Scherrer equation.
