# Supplementary material for: Comparison of ZrO2 Particles and Polyaniline as Additives in Polystyrene-Based Sorbents for the Micro-Solid Phase Extraction of Psychoactive Drugs from Biofluids
Source: Molecules. 2024 Feb 7;29(4):761. doi: 10.3390/molecules29040761 (PMC10893364; doi:10.3390/molecules29040761)
Supplement: Supplementary file 1 [file molecules-29-00761-s001.zip › molecules-2852125-supplementary.pdf]

Comparison of ZrO_2 particles and polyaniline as additives in polystyrene-based sorbents for the micro-solid phase extraction of psychoactive drugs from biofluids

Paweł Stelmaszczyk [1,2], Mateusz Iwan [1], Dominika Pawcenis [3] and Renata Wietecha-Posłuszny [1,]*

[1] Laboratory for Forensic Chemistry, Department of Analytical Chemistry, Faculty of Chemistry, Jagiellonian University in Kraków, Gronostajowa St. 2, 30-387 Kraków, Poland; pawel.stelmaszczyk@doctoral.uj.edu.pl (P.S.); mateusz.iwan@alumni.uj.edu.pl (M.I.)
[2] Doctoral School of Exact and Natural Sciences, Faculty of Chemistry, Jagiellonian University in Kraków, Gronostajowa St. 2, 30-387 Kraków, Poland
[3] Faculty of Chemistry, Jagiellonian University in Kraków, Gronostajowa St. 2, 30-387 Kraków, Poland; pawcenis@chemia.uj.edu.pl
* **Correspondence: wietecha@chemia.uj.edu.pl or renata.wietecha-posluszny@uj.edu.pl**

Keywords: electrospun fibers, solid phase microextraction, sorbents, polystyrene, psychoactive substances

Table of contents:

1. XRD pattern and SEM image of synthesized ZrO_2 particles
2. Calculated diameter of synthesized ZrO_2 particles based on the XRD pattern

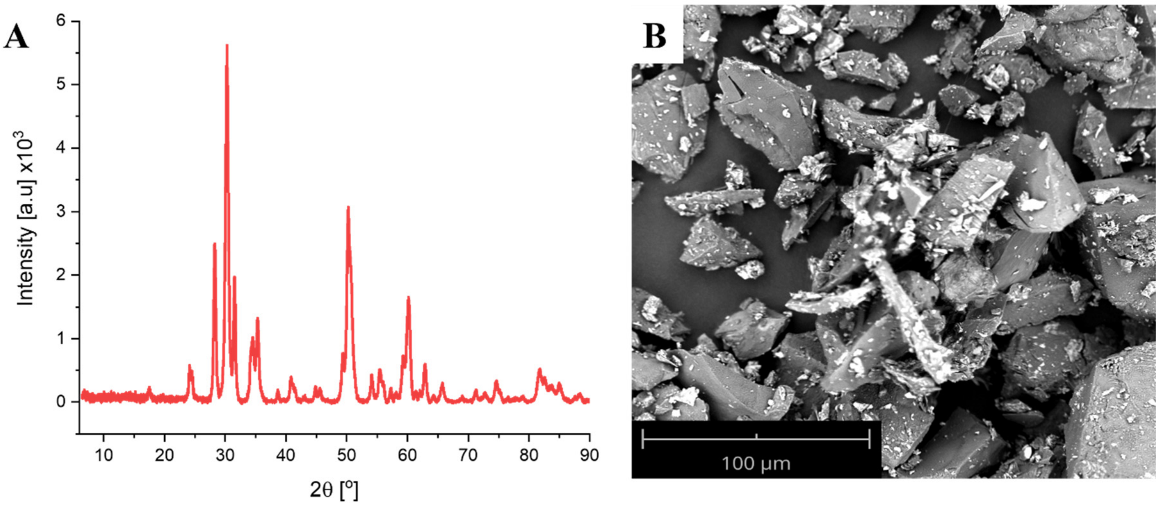

Figure S1. A – XRD pattern (λ = 1.5405 Å, Bragg-Brentano geometry, 2θ range: 5-90°) of synthesized ZrO_2 particles; B – SEM image of obtained ZrO_2 particles. Both XRD pattern and SEM image were obtained for ZrO_2 particles before calcination or washing with HNO_3.

Table S1. The calculated diameter of synthesized ZrO_2 particles based on the XRD pattern and Scherrer equation.

**2θ (°)**	**θ (°)**	***B* (rad)**	**D_{hkl} (nm)**
28.2	14.1	0.006801	21.02
30.3	15.1	0.009339	15.38
31.5	15.8	0.006840	21.06
34.5	17.3	0.014770	9.83
35.3	17.7	0.009145	15.91
50.3	25.1	0.015330	9.99
60.1	30.1	0.011830	13.54
